# Insulin-Like Growth Factor 1 on the Maintenance of Ribbon Synapses in Mouse Cochlear Explant Cultures

**DOI:** 10.3389/fncel.2020.571155

**Published:** 2020-10-08

**Authors:** Li Gao, Tomoko Kita, Tatsuya Katsuno, Norio Yamamoto, Koichi Omori, Takayuki Nakagawa

**Affiliations:** Department of Otolaryngology, Head and Neck Surgery, Graduate School of Medicine, Kyoto University, Kyoto, Japan

**Keywords:** cochlea, insulin-like growth factor 1, inner hair cell, ribbon synapse, maintenance

## Abstract

Hearing loss has become one of the most common disabilities worldwide. The synaptic connections between inner hair cells (IHCs) and spiral ganglion neurons have specialized synaptic constructions, termed ribbon synapses, which are important for auditory function. The ribbon synapses in the cochlea are quite vulnerable to various insults. As such, the maintenance of ribbon synapses is important for ensuring hearing function. Insulin-like growth factor 1 (IGF1) plays a critical role in the development and maintenance of the cochlea and has the potential to protect cochlear hair cells from various insults. In this study, we examined the role of IGF1 in the maintenance of ribbon synapses in cochlear explants of postnatal day four mice. We cultured cochlear explants with an IGF1 receptor antagonist, JB1, which is an IGF1 peptide analog. Results showed that exposure to JB1 for 24 h resulted in the loss of ribbon synapses. After an additional 24-h culture without JB1, the number of ribbon synapses spontaneously recovered. The application of exogenous IGF1 showed two different aspects of ribbon synapses. Low doses of exogenous IGF1 promoted the recovery of ribbon synapses, while it compromised the spontaneous recovery of ribbon synapses at high doses. Altogether, these results indicate that the paracrine or autocrine release of IGF1 in the cochlea plays a crucial role in the maintenance of cochlear ribbon synapses.

## Introduction

Hearing loss has become one of the most common disabilities worldwide. The World Health Organization (2018) estimates that hearing loss affects over 6.1% (around 466 million people) of the world’s population. The cochlea is the sensory organ responsible for auditory function. Hearing is a series of events in which sound vibrations travel into the cochlea and are then converted into electrochemical signals that are perceived by the auditory cortex. In the mammalian cochlea, the conversion is processed by mechanosensory hair cells (HCs) consisting of outer and inner hair cells (IHCs). Outer hair cells (OHCs) act as motor units that amplify the sound stimuli and contact type II spiral ganglion neurons (SGNs). IHCs play a key role in the process of auditory transduction. IHCs contact type I SGNs and convey electrochemical signals to the central auditory system (Hudspeth, 1997). Synaptic connections between IHCs and type I SGNs have specialized synaptic constructions, termed ribbon synapses, and are important for auditory function (Safieddine et al., 2012; Coate et al., 2019).

The ribbon synapse is an electron-dense structure associated with neurotransmitter-filled vesicles at sensory synapses in the retina, lateral line, and inner ear organs (Becker et al., 2018). Unlike conventional synapses, the ribbon synapse is specialized for the transmission of sensory information. The rapid and sustained neurotransmitter release from ribbon synapses allows for the proper transfer of sensory information to the postsynaptic receptors (Safieddine et al., 2012). The ribbon synapses in the cochlea are highly vulnerable to various insults such as ototoxic agents (Liu et al., 2013) and noise-induced injury (Kujawa and Liberman, 2009). Recent studies have demonstrated the associations between the loss of ribbon synapses and difficulties in understanding speech in noisy environments (Bharadwaj et al., 2014) and age-related hearing impairment (Viana et al., 2015). Therefore, the maintenance of ribbon synapses is crucial for ensuring normal hearing function. This encourages investigation into the protection and regeneration of cochlear ribbon synapses. Both a TrkB agonist (Meltser et al., 2014) and an AMPA receptor blocker (Hu et al., 2020) have demonstrated the protection of cochlear ribbon synapses against noise trauma. In an aminoglycoside-induced degeneration model, the protection of cochlear ribbon synapses by the local application of fibroblast growth factor 22 has also been reported (Li et al., 2016). Although still controversial (Wagner and Shin, 2019), the capacity to regenerate ribbon synapses in the mammalian cochlea was demonstrated *in vitro* (Wang and Green, 2011; Yamahara et al., 2019) and *in vivo* (Wan et al., 2014; Suzuki et al., 2016).

Insulin-like growth factor 1 (IGF1), once known as somatomedin C, is a hormone similar in molecular structure to insulin. IGF1 is essential for controlling cell proliferation, differentiation, and apoptosis in various tissues and organs, including the inner ears (Varela-Nieto et al., 2007; Okano et al., 2011; Allahdadi et al., 2019). IGF1 has the potential to protect HCs from excessive noise (Iwai et al., 2006; Lee et al., 2007), aminoglycoside antibiotics (Hayashi et al., 2017), ischemic trauma (Fujiwara et al., 2008), and surgical invasion (Yamahara et al., 2018). Also, the therapeutic effects of recombinant human IGF1 (rhIGF1) on sudden sensorineural hearing loss refractory to systemic corticosteroids have been confirmed in clinical studies (Nakagawa et al., 2010, 2014, 2016). IGF1 performs its functions by binding to its tyrosine kinase receptor (IGF1R). In the cochlea, IGF1R is expressed on the surface of OHCs, IHCs, and all supporting cells, including pillar cells, Hensen’s and Claudius’ cells, inner sulcus cells, and Deiters’ cells (Okano et al., 2011; Hayashi et al., 2013), as well as SGNs (Sanchez-Calderon et al., 2010). Our previous study confirmed that IGF1 induced the regeneration of ribbon synapses in cochlear explant cultures of postnatal day (P) two mice (Yamahara et al., 2019). In this study, we examined the roles of IGF1 signaling in the maintenance of cochlear ribbon synapses using explant cultures. For the pharmacological inhibition of IGF1Rs, we used a competitive IGF1R antagonist, JB1, which is an IGF1 peptide analog. JB1 is a 12-amino acid cyclic peptide that inhibits the binding of IGF1 to its receptor (Pietrzkowski et al., 1992). We further confirmed the potential for spontaneous recovery of ribbon synapses and the additional effect of exogenous IGF1 in cochlear explants that had previously been damaged by JB1.

## Materials and Methods

### Animals

Institute of Cancer Research (ICR) mice aged postnatal day 4 were purchased from Japan SLC (Hamamatsu, Japan). All animal procedures were performed following the NIH Guide for the Care and Use of Laboratory Animals (NIH Publications No. 8023, revised 1978) and were approved by the Animal Research Committee of Kyoto University Graduate School of Medicine (Med Kyo 17,114, 18,117, 19,536).

### Explant Cultures

Cochlear explant cultures were performed as previously described (Yamahara et al., 2019). Briefly, P4 mice were killed with carbon dioxide (CO_2_), after which they were decapitated. The cochleae were removed from the temporal bones and dissected in ice-cold sterile 0.1 M phosphate-buffered saline (PBS). The surrounding bony capsules were removed, and cochlear sensory epithelia were carefully dissected to preserve afferent connections with SGNs. To minimize any differences related to the region of cochlea under study, we only used the middle portion (40–60% from the apex) of the cochlear sensory epithelia. The samples were placed on cell culture inserts (Becton Dickinson, Franklin Lake, NJ, USA) and incubated in Dulbecco’s modified Eagle’s medium (DMEM; Sigma–Aldrich Inc., St. Louis, MO, USA) supplemented with 6 g/l D-glucose (Wako Pure Chemicals, Osaka, Japan) and 0.15 g/l penicillin G (Wako Pure Chemicals, Osaka, Japan) at 37°C in a humidified atmosphere with 5% CO_2_. To prevent any effects caused by serum growth factors or hormones, serum-free medium was used in all experiments. Overnight pre-cultures were established to stabilize the cochlear explants.

### Pharmacological Inhibition by JB1

After the overnight pre-cultures, cochlear explants were exposed to JB1 (Cat. No. J3705; Sigma–Aldrich Inc., St. Louis, MO, USA) at a concentration of 25, 50, 100, or 150 μg/ml for 24 h (*n* = 5 for each). Cochlear explants that were cultured without exposure to JB1 were used as controls. Cultured samples were histologically assessed for the number of IHCs, density of SGNs, as well as number of density of SGNs, as well as number of presynaptic ribbons, and postsynaptic receptor patches per IHC.

### Spontaneous Recovery of Ribbon Synapses

To assess the capacity for spontaneous recovery of ribbon synapses, we used cochlear explants that were pre-treated with 50 μg/ml JB1 for 24 h. After rinsing for 5 min thrice with culture media, cochlear explants (*n* = 9) were incubated in the culture media for another 24 h without being exposed to JB1. The cochlear explants exposed to JB1 for 24 h were used as controls (*n* = 9). The number of presynaptic ribbons and postsynaptic receptor patches per IHC was compared between the two groups.

### Effects of Exogenous IGF1 on Ribbon Synapses

To determine the effect of exogenous IGF1 on the recovery of presynaptic ribbons and postsynaptic receptor patches, cochlear explants that had been exposed to 50 μg/ml JB1 for 24 h were incubated with culture media supplemented with rhIGF1 (Orphan Pacific Pharma, Tokyo, Japan) at a concentration of 0, 0.1, 0.15, 0.2, 0.5, 1.0, or 5.0 μg/ml (*n* = 5–10) for 24 h. The specimens were then histologically examined.

### Immunohistochemistry

Specimens were fixed in 4% paraformaldehyde in phosphate buffer (0.1 M; pH 7.4) for 15 min at room temperature (RT) and then rinsed with PBS. We analyzed ribbon synapses by immunohistochemical staining for C-terminal-binding protein 2 (CtBP2) and ionotropic glutamate receptor-2 (GluA2), as previously described (Liberman and Liberman, 2016; Suzuki et al., 2016). After blocking with 10% goat serum in 0.2% Triton™ X-100 for 30 min at RT, we incubated the specimens at 37°C overnight with the primary antibodies shown in Table 1 diluted in 10% goat serum in PBS. We incubated samples with primary antibody species-specific secondary antibodies with Alexa Fluor^®^ conjugates (Table 1) for 1 h at RT.

**Table 1:** The information of antibodies.

Antibody	Working dilution	Cat. No.	Company
Rabbit polyclonal anti-myosin 7a	1:500	25-6790	Proteus BioScience, Ramona, CA, USA
Mouse monoclonal immunoglobulin G (IgG)1 anti-CtBP2	1:250	612044	BD Biosciences, Franklin Lakes, NJ, USA
Mouse monoclonal IgG2a anti-GluA2	1:2,000	MAB397	Merck Millipore, Burlington, MA, USA
Chicken polyclonal anti-neurofilament-H	1:1,000	AB5539	Merck Millipore, Burlington, MA, USA
Alexa Fluor 405 Goat anti-rabbit lgG (H+L)	1:1,000	A-31556	Thermo Fisher Scientific, Waltham, MA, USA
Alexa Fluor 568 Goat anti-mouse lgG1	1:1,000	A-21124	Thermo Fisher Scientific, Waltham, MA, USA
Alexa Fluor 488 Goat anti-mouse lgG2a	1:1,000	A-21131	Thermo Fisher Scientific, Waltham, MA, USA
Alexa Fluor 647 Goat anti-chicken lgG (H+L)	1:1,000	A-21449	Thermo Fisher Scientific, Waltham, MA, USA

### Presynaptic Ribbon and Postsynaptic Receptor Patch Counting

Cochlear specimens were imaged with a Leica TCS-SPE confocal microscope (Leica Microsystems, Wetzlar, Germany) equipped with 405, 488, 532, and 635 nm solid-state lasers. Scanning was sequential with a 2.5 μs dwell time. Images were sampled at a resolution of 512 by 512 pixels with a 63× oil immersion (NA 1.3) objective, a 1.5× software zoom, and a *z*-step size of 0.17 μm. A series of images contained at least 10 IHCs from the cuticular plate to the base. For SGN density analysis, a *z-step* size of 0.5 μm was applied to include the soma and SGN afferent dendrites. Imaris 7.4.2 software (BitPlane AG, Zurich, Switzerland) was used to perform three-dimensional reconstructions and spot analyses to count the number of presynaptic ribbons and postsynaptic receptor, as previously described (Fogarty et al., 2013). Puncta in each IHC with immunoreactivity for CtBP2 were counted as presynaptic ribbons. Puncta in each IHC with immunoreactivity for both CtBP2 and GluA2 were defined as postsynaptic receptor patches. For the spot analysis, the minimum diameter of a punctum was defined as 0.7 μm. For each sample, the number of presynaptic ribbons and postsynaptic receptor patches was manually counted in 10 IHCs.

### Statistical Analysis

Student’s *t*-test or one-way analysis of variance (ANOVA) with Tukey *post hoc* test was used. Statistical analysis was performed using GraphPad Prism v6.0 for Mac (GraphPad, San Diego, CA, USA) and SPSS 25 version for Mac (SPSS Inc., Chicago, IL, USA). The data are expressed as the mean ± standard deviation. Differences with *p-values* <0.05 were considered statistically significant.

## Results

### An IGF1R Antagonist Induced Loss of Ribbon Synapses in Cochlear Explant Cultures

**Figure 1:**
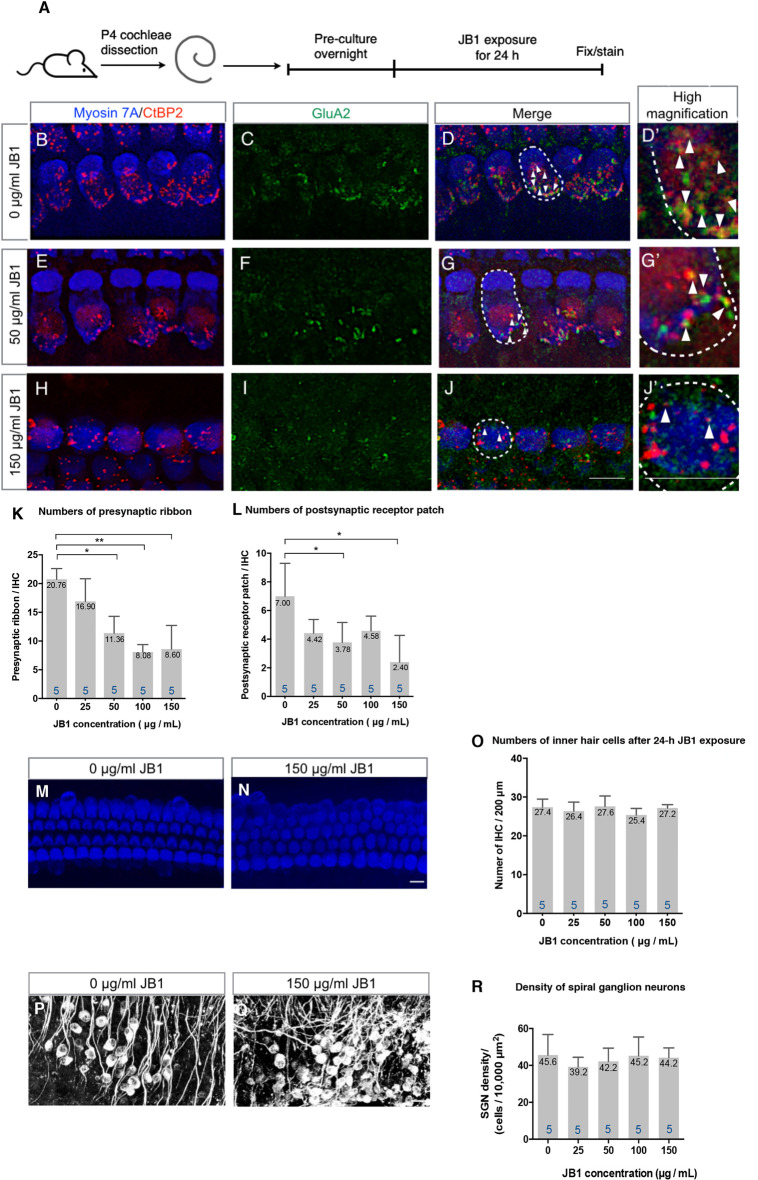
An insulin-like growth factor 1 receptor (IGF1R) antagonist JB1 induced the loss of ribbon synapses in cochlear explant cultures. Cochlear explants of P4 mice were exposed to JB1 at a concentration of 25, 50, 100, or 150 μg/ml for 24 h **(A)**. **(B–D)** Maximal-intensity projection images with *z-stack* of ribbon synapses in control specimens that were cultured without JB1. Panels **(E–J)** are maximal-intensity projection images of the degeneration of ribbon synapses in specimens cultured with JB1 at concentrations of 50 and 150 μg/ml, respectively. Arrows show the postsynaptic receptor patches and dotted lines indicate the location of an IHC. **(K)** JB1 showed significant effects on both presynaptic ribbons (*p* < 0.001 by one-way ANOVA) and postsynaptic receptor patches (**L**; *p* = 0.004 by one-way ANOVA). Tukey’s *post hoc* test revealed significant losses at concentrations of 50 (*p* = 0.001), 100 (*p* < 0.001), and 150 μg/ml (*p* < 0.001) in the number of presynaptic ribbons, and at concentrations of 50 (*p* = 0.032) and 150 μg/ml (*p* = 0.002) in the number of postsynaptic receptor patches, in comparison with controls which were cultured without JB1. **(M–R)** No inner hair cell (IHC) or spiral ganglion neuron (SGN) loss was found at any JB1 concentrations (*p* = 0.428 by one-way ANOVA for IHC, *p* = 0.730 by one-way ANOVA for SGN). Scale bars: 10 μm. Data are expressed as mean (digits at the top of each bar) ± SD. The digits at the bottom of each bar represent the sample number. *p < 0.05; **p < 0.01 with Tukey’s *post hoc* test. P4, postnatal day 4; IHC, inner hair cell; SGN, spiral ganglion neuron; SD, standard deviation; ANOVA, analysis of variance.

To assess the effect of pharmacological inhibition of IGF1R on the maintenance of cochlear ribbon synapses, we exposed cochlear explant cultures of P4 mice to an IGF1R antagonist, JB1, at different concentrations, ranging from 0 to 150 μg/ml, for 24 h (Figure 1A). All assays were performed using the middle portion (40–60% from the apex) of the cochlea because the numbers of ribbon synapses per IHC varied according to the location in the cochlear turns (Meyer et al., 2009). Immunohistochemistry for CtBP2 for labeling presynaptic ribbons and GluA2 for labeling postsynaptic receptor patches was used to assess the ribbon synapses histologically. The location of IHCs was determined by immunostaining for myosin VIIa. Immunohistochemical analyses for CtBP2 and GluA2 demonstrated ribbon synapses in control specimens that were cultured without JB1 (Figures 1B–D). A decrease in the number of ribbon synapses was observed in specimens cultured with JB1 at concentrations of 50 (Figures 1E–G) and 150 μg/ml (Figures 1H–J). We quantified the number of CtBP2-positive puncta (as presynaptic ribbons) and CtBP2 and GluA2 co-stained puncta (as postsynaptic receptor patches) per IHC, respectively. JB1 had significant effects on both presynaptic ribbons (*p* < 0.001 by one-way ANOVA) and postsynaptic receptor patches (*p* = 0.004 by one-way ANOVA), respectively (Figures 1K,L). Tukey’s *post hoc* test revealed significant losses at concentrations of 50 (*p* = 0.001), 100 (*p* < 0.001) and 150 μg/ml (*p* < 0.001) in the number of presynaptic ribbons, and at concentrations of 50 (*p* = 0.032) and 150 μg/ml (*p* = 0.002) in the number of postsynaptic receptor patches, in comparison with controls which were cultured without JB1 (Figures 1K,L). No significant loss of IHC (Figures 1M–R) or SGN (Figures 1P–R) was found at any JB1 concentration (*p* = 0.428 by one-way ANOVA for IHC, *p* = 0.730 by one-way ANOVA for SGN). These findings demonstrate that the pharmacological inhibition of IGF1R caused a decrease in both presynaptic ribbons and postsynaptic receptor patches, suggesting that IGF1 is necessary for the maintenance of ribbon synapses in cochlear explant cultures.

### Spontaneous Recovery of Ribbon Synapses

**Figure 2:**
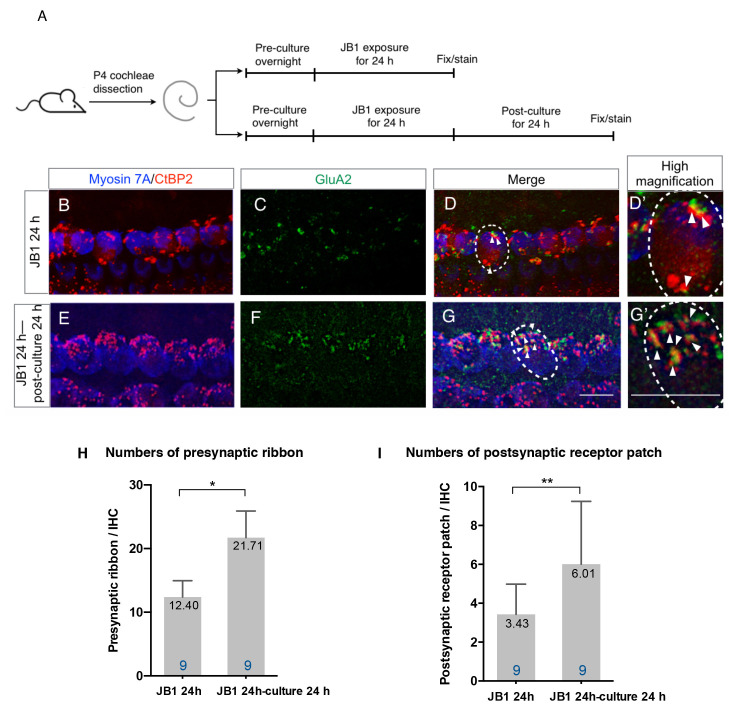
Spontaneous recovery of ribbon synapses. Cochlear explants from P4 mice were exposed to 50 μg/ml JB1 for 24 h, with or without an additional 24 h of culture **(A)**. **(B–D)** Maximal-intensity projection images with *z*-stack of the immunostaining results of specimens immediately after 24-h JB1 exposure. **(E–G)** Maximal-intensity projection images of the specimens following an additional 24-h culture. Arrows show the postsynaptic receptor patches and dotted lines indicate the location of an IHC. **(H)** The number of presynaptic ribbons (*p* < 0.001 by unpaired *t*-test) and postsynaptic receptor patches (**I**; *p* = 0.046 by unpaired *t*-test) in the specimens were significantly increased after an additional 24-h culture. Scale bars: 10 μm. Data are expressed as mean (digits at the top of each bar) ± SD. The digits at the bottom of each bar represent the sample number. *p < 0.01; **p < 0.05 by an unpaired *t*-test. P4, postnatal day 4; IHC, inner hair cell; SD, standard deviation.

To test the spontaneous recovery capacity of ribbon synapses, we performed an additional 24-h culture without the presence of JB1 following a 24-h exposure period to JB1 at a concentration of 50 μg/ml (Figure 2A). Compared with cochlear explants exposed to JB1 for 24 h (Figures 2B–D), cochlear explants incubated for an additional 24 h without being exposed to JB1 tended to recover their ribbon synapses (Figures 2E–G). Statistical analyses using unpaired *t*-test demonstrated a significant increase in the numbers of both presynaptic ribbons (Figure 2H, *p* < 0.001, student’s *t*-test) and postsynaptic receptor patches (Figure 2I, *p* = 0.046, student’s *t*-test) in specimens following an additional 24-h culture. These findings indicate that cochlear explants of P4 mice have the capacity for spontaneous recovery of both presynaptic ribbons and postsynaptic receptor patches following their loss because of the pharmacological inhibition of IGF1R.

### Effects of Exogenous IGF1 on the Recovery of Cochlear Ribbon Synapses

**Figure 3:**
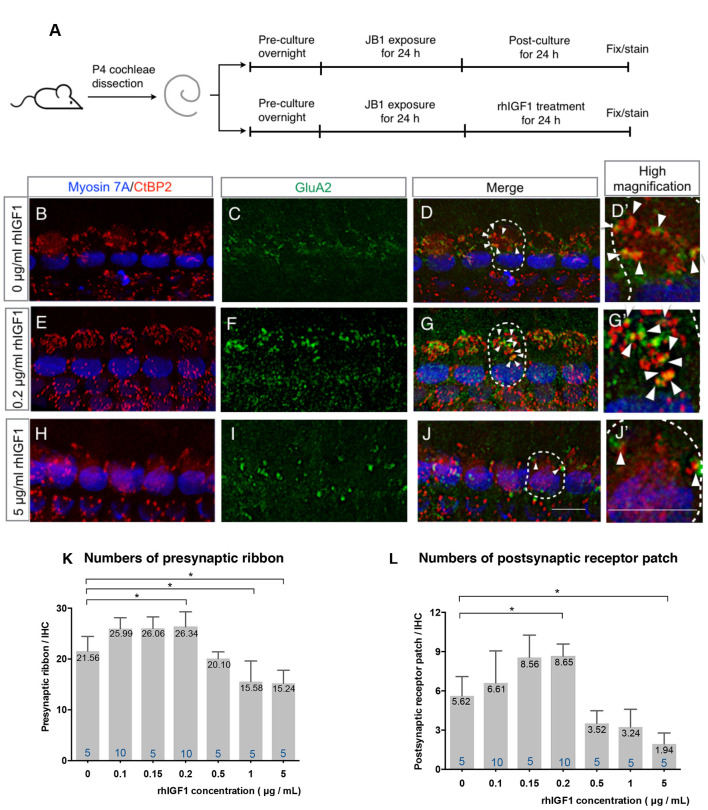
Effect of exogenous IGF1 on the recovery of ribbon synapses. Cochlear explants from P4 mice were exposed to 50 μg/ml JB1 for 24 h, after which they were incubated with culture media supplemented with rhIGF1 at a concentration of 0.1, 0.15, 0.2, 0.5, or 1, 5 μg/ml for 24 h **(A)**. **(B–D)** Maximal-intensity projection images with *z*-stack of the immunostaining images of specimens cultured without rhIGF1. Panels **(E–J)** are maximal-intensity projection images of the recovery of ribbon synapses in specimens cultured with 50 μg/ml JB1 for 24 h, followed by rhIGF1 treatment for 24 h at concentrations of 0.2 and 5 μg/ml, respectively. Arrows show the postsynaptic receptor patches and dotted lines indicate the location of an IHC. Exogenous rhIGF1 showed significant effects on the number of both presynaptic ribbons (**K**; *p* < 0.001 by one-way ANOVA) and postsynaptic receptor patches (**L**; *p* < 0.001 by one-way ANOVA). Tukey’s *post hoc* test revealed significant difference at concentrations of 0.2 (*p* = 0.034), 1 (*p* = 0.017), and 5 μg/ml (*p* = 0.010) in the number of presynaptic ribbons, and at concentrations of 0.2 (*p* = 0.020) and 5 μg/ml (*p* = 0.012) in the number of postsynaptic receptor patches, in comparison with controls which were cultured in 0 μg/ml rhIGF1. Scale bars: 10 μm. Data are expressed as mean (digits at the top of each bar) ± SD. The digits at the bottom of each bar represent the sample number. *p < 0.05 by one-way ANOVA, followed by Tukey’s *post hoc* test. P4, postnatal day 4; IHC, inner hair cell; SD, standard deviation; rhIGF1, recombinant human insulin-like growth factor-1; ANOVA, analysis of variance.

To assess the additional effects of exogenous IGF1 on the recovery of ribbon synapses, cochlear explants that had been damaged by 24-h exposure to 50 μg/ml JB1 were incubated with culture media supplemented with rhIGF1 for 24 h (Figure 3A). Compared with the specimens following an additional 24-h culture without supplementation of rhIGF1 (Figures 3B–D), further increase in the number of ribbon synapses was observed in the specimens following an additional 24-h culture with 0.2 μg/ml rhIGF1 (Figures 3E–G). However, in those following an additional 24-h culture with 5 μg/ml rhIGF1 (Figures 3H–J), a decrease in the number of ribbon synapses was observed. Quantitative analyses showed significant effects of exogenous rhIGF1 on the numbers of both presynaptic ribbons (*p* < 0.001, one-way ANOVA, Figure 3K) and postsynaptic receptor patches (*p* < 0.001, one-way ANOVA, Figure 3L). Significant differences were identified in the number of presynaptic ribbons between 0 and 0.2 (*p* = 0.034, Tukey *post hoc* test), 1 (*p* = 0.017, Tukey *post hoc* test) or 5 μg/ml (*p* = 0.010, Tukey *post hoc* test), and in the number of postsynaptic receptor patches between 0 and 0.2 (*p* = 0.020, Tukey *post hoc* test) or 5 μg/ml (*p* = 0.012, Tukey *post hoc* test). In summary, exogenous IGF1 at concentrations <0.5 μg/ml exerted positive effects on the recovery of ribbon synapses, but high concentrations diminished spontaneous recovery. We tested the effect of rhIGF1 at a concentration of 5 μg/ml on the survival of IHCs or SGNs. The results demonstrated no significant IHC or SGN loss in specimens treated with 5 μg/ml rhIGF1 compared with those cultured without rhIGF1 (Supplementary Figure S1). These findings indicate that the precise regulation of IGF1 levels may be required for the maintenance of ribbon synapses in cochlear explant cultures.

## Discussion

In this study, we used the IGF1R antagonist JB1 to pharmacologically inhibit IGF1 signaling. JB1 is a highly selective IGF-IR antagonist, which interferes with the efficacy of IGF1 by competitively inhibiting the binding of IGF1 to IGF1R (Pietrzkowski et al., 1992). Our previous study demonstrated the efficacy of JB1 in attenuating IGF1-mediated regeneration of ribbon synapses in P2 cochlear explants that had been damaged by excitatory amino acids (Yamahara et al., 2019). JB1 has also been used to antagonize IGF1 effects in various organs including the hippocampus (Nelson et al., 2014) and retina (Landi et al., 2009). The present results demonstrated that JB1 decreased the number of both presynaptic ribbons and postsynaptic receptor patches in a dose-dependent manner, indicating that IGF1 is an inevitable factor for the maintenance of ribbon synapses in cochlear explants. Our previous study (Yamahara et al., 2019) used explant cultures of P2 mouse cochleae, while in the present study, we used P4 mouse cochleae for a couple of reasons. One is the maturation process of ribbon synapses. The period from P0 to P12 is an active phase for the maturation of ribbon synapses (Michanski et al., 2019), indicating that ribbon synapses in P4 mice are more mature than those in P2 mice. On the other hand, there is a technical limitation in the preparation of cochlear explant culture. It is difficult to dissect out cochlear sensory epithelia from P5 or older mouse cochleae. Based on these issues, P4 mice were chosen for this study. Ribbon synapses in P4 mouse cochleae are still immature, which can be a limitation of the present study.

Previous studies have revealed the importance of IGF1 for the maturation of cochleae after birth (Camarero et al., 2001, 2002). In homozygous mutants, IGF1^−/−^ mice, no significant loss of SGNs was identified at P5, while SGN numbers and sizes were statistically significantly decreased at P20. Moreover, cochlear volumes were also revealed to have the same trend (Camarero et al., 2001). Abnormal innervation of SGN afferent dendrites was observed at P20, but not at P5 (Camarero et al., 2002). Also, IGF1-deficient mice presented profound deafness from 1 month of age onwards, without any obvious worsening of hearing parameters with aging (Riquelme et al., 2010). These findings suggest the importance of IGF1 for the maintenance and maturation of the synaptic contacts between IHCs and SGNs.

IGF1 is mainly produced in the liver and exerts its function through endocrine, autocrine, and paracrine mechanisms (Jenkins and Bustin, 2004). IGF1 is also synthesized in the developing and neonatal cochleae (Sanchez-Calderon et al., 2010; Okano et al., 2011), indicating the existence of paracrine or autocrine systems for IGF1 in the cochlea. In this study, we observed the spontaneous recovery of both presynaptic ribbons and postsynaptic patches after their loss because of the pharmacological inhibition of IGF1R. Therefore, we presume that the paracrine or autocrine systems in the cochlea may contribute to the spontaneous recovery of ribbon synapses in cochlear explants that had been damaged by JB1.

Neurotrophin-3 (NT-3) is a potent factor for the maintenance and regeneration of cochlear ribbon synapses (Wang and Green, 2011; Wan et al., 2014). The selective blockade of endogenous NT-3 signaling reduced the regeneration of axons and postsynaptic densities *in vitro* (Wang and Green, 2011). Endogenous NT-3 promoted the recovery of cochlear ribbon synapses after acoustic trauma *in vivo* (Wan et al., 2014). Brain-derived neurotrophic factor (BDNF) also plays a role in the development and maintenance of ribbon synapses (Zuccotti et al., 2012; Meltser et al., 2014; Wan et al., 2014). In this study, the selective blockade of IGF1R induced the significant loss of cochlear ribbon synapses, suggesting that endogenous NT-3 or BDNF in P4 cochlear explants could not compensate for the loss of IGF1 signaling. In contrast, other factors, including NT-3 and BDNF, may be involved in the process of spontaneous recovery of ribbon synapses that were observed in this study.

In our previous studies using cochlear explants of neonatal mice, exogenous rhIGF1 had protective effects against aminoglycoside ototoxicity on IHCs at concentrations ranging from 0.1 to 1 μg/ml, and on OHCs at concentrations ranging from 10^−4^ to 1 μg/ml (Hayashi et al., 2013; Yamahara et al., 2017). For the regeneration of ribbon synapses after excitotoxic insults, 1 μg/ml exogenous rhIGF1 showed significant effects, but 5 μg/ml did not (Yamahara et al., 2019). Based on these findings, we used exogenous rhIGF1 at concentrations ranging from 0.1 to 5 μg/ml in this study. These results demonstrated the positive effects on the recovery of both presynaptic ribbons and postsynaptic receptor patches at concentrations of rhIGF1 ranging from 0.1 to 0.2 μg/ml, and negative effects at a concentration of 1 and 5 μg/ml. Compared with our previous study using cochlear explants that were damaged by excitatory amino acids (Yamahara et al., 2019), the concentration of rhIGF1 required to promote recovery is lower, which could be due to differences in the method used to induce damage. In contrast to the present results, after the exposure to excitatory amino acids, no spontaneous recovery of ribbon synapses was observed, indicating the lack of endogenous IGF1 production. In the present study, exogenous rhIGF1 exhibited bell-shaped dose-response curves in the numbers of presynaptic ribbons and postsynaptic receptor patches. This type of dose-response curve is common with endocrine disruption (Vandenberg et al., 2012). Some hormones, including androgen and estradiol, show agonist effects at low concentrations and antagonist effects at high concentrations. There are several mechanisms for a bell-shaped dose-response curve, including actions at multiple targets, cytotoxicity, receptor selectivity, receptor down-regulation and desensitization, receptor competition, and endocrine negative feedback loops (Vandenberg et al., 2012). IGF1 is similar in molecular structure to insulin and has a low affinity to insulin receptors. At high concentrations, IGF1 activates insulin receptor-mediated signaling. Altogether, the precise regulation of IGF1 levels may be necessary for the maintenance of cochlear ribbon synapses.

In conclusion, in the current study, we demonstrated that the pharmacological inhibition of IGF1R reduced the number of ribbon synapses in cochlear explant cultures of P4 mice, pointing to the importance of IGF1 for the maintenance of ribbon synapses. Surprisingly, the number of ribbon synapses recovered spontaneously following an additional 24 h of culture, pointing to the contribution of endogenous IGF1 to the maintenance of cochlear ribbon synapses. This confirmation of the effects of exogenous rhIGF1 points to the precise regulation of IGF1 levels for the maintenance of cochlear ribbon synapses. However, ribbon synapses in P4 cochleae are still in their maturation process (Michanski et al., 2019), which is a limitation of this study. We will investigate the role of IGF1 in the maintenance of cochlear ribbon synapses in mature animals *in vivo* shortly.

## Data Availability Statement

The raw data supporting the conclusions of this article will be made available by the authors, without undue reservation.

## Ethics Statement

The animal study was reviewed and approved by the Animal Research Committee of Kyoto University Graduate School of Medicine.

## Author Contributions

TN designed the study. LG, TKi and TKa performed the experiments. LG and TN analyzed the data and wrote the manuscript. NY and KO performed a critical review.

## Conflict of Interest

The authors declare that the research was conducted in the absence of any commercial or financial relationships that could be construed as a potential conflict of interest.

## Supplementary Material

FIGURE S1 Effects of exogenous IGF1 on the survival of inner hair cells (IHCs) and spiral ganglion neurons (SGNs). Cochlear explants from P4 mice were exposed to 50 μg/ml JB1 for 24 h, after which they were incubated with culture media supplemented with rhIGF1 at a concentration of 5 μg/ml or without rhIGF1 for 24 h. **(A–D)** Maximal-intensity projection images with *z*-stack of the immunostaining images of specimens cultured with rhIGF1 at concentrations of 0 and 5 μg/ml. **(E,F)** No significant loss of inner hair cell (IHC) or spiral ganglion neuron (SGN) was found with student’s *t*-test (*p* = 0.578 for IHC and *p* = 0.133 for SGN). Scale bars: 10 μm. Data are expressed as mean (digits at the top of each bar) ± SD. The digits at the bottom of each bar represent the sample number.

## Abbreviations

BDNF: brain-derived neurotrophic factor
CtBP2: C-terminal-binding protein 2
HC: hair cell
IGF1: Insulin-like growth factor 1
IGF1R: Insulin-like growth factor 1 receptor
IHC: Inner hair cell
NT-3: Neurotrophin-3
OHC: Outer hair cell
P: Postnatal day
PBS: phosphate-buffered saline
rhIGF1: Recombinant human insulin-like growth factor 1
RT: Room temperature
SGN: Spiral ganglion neuron.
