# Supplementary figures and images for: Insulin-Like Growth Factor 1 on the Maintenance of Ribbon Synapses in Mouse Cochlear Explant Cultures
Source: Front Cell Neurosci. 2020 Oct 8;14:571155. doi: 10.3389/fncel.2020.571155 (PMC7579230; doi:10.3389/fncel.2020.571155)

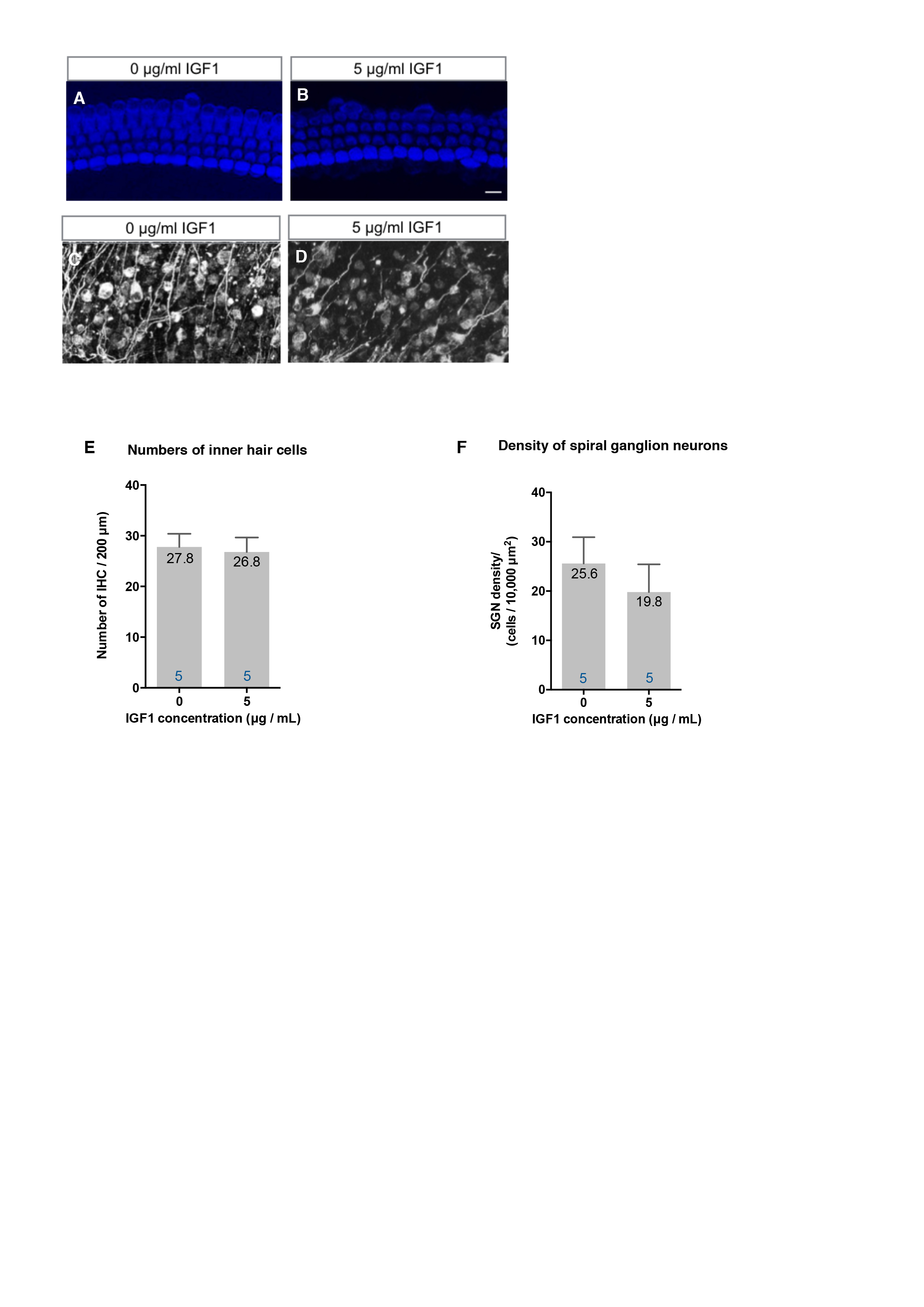

Supplement: FIGURE S1 — Effects of exogenous IGF1 on the survival of inner hair cells (IHCs) and spiral ganglion neurons (SGNs). Cochlear explants from P4 mice were exposed to 50 μg/ml JB1 for 24 h, after which they were incubated with culture media supplemented with rhIGF1 at a concentration of 5 μg/ml or without rhIGF1 for 24 h. (A–D) Maximal-intensity projection images with z-stack of the immunostaining images of specimens cultured with rhIGF1 at concentrations of 0 and 5 μg/ml. (E,F) No significant loss of inner hair cell (IHC) or spiral ganglion neuron (SGN) was found with student’s t-test (p = 0.578 for IHC and p = 0.133 for SGN). Scale bars: 10 μm. Data are expressed as mean (digits at the top of each bar) ± SD. The digits at the bottom of each bar represent the sample number. [file Image_1.JPEG]
